# Development and Validation of a Cytokine‐Based Predictive Model for Acute GvHD and Composite Outcomes in ATG–Based Haploidentical Hematopoietic Stem Cell Transplantation

**DOI:** 10.1155/mi/3165406

**Published:** 2026-06-30

**Authors:** Yiyin Chen, Xinghao Yu, Zhou Jin, Chuanhe Jiang, Xiaoxia Hu, Yang Xu

**Affiliations:** ^1^ National Clinical Research Center for Hematologic Diseases, Jiangsu Institute of Hematology, Jiangsu Key Laboratory of Hematologic Diseases, The First Affiliated Hospital of Soochow University, Suzhou, Jiangsu, China, sdfyy.cn; ^2^ Institute of Blood and Marrow Transplantation, Collaborative Innovation Center of Hematology, Soochow University, Suzhou, Jiangsu, China, scu.edu.tw; ^3^ Department of Medicine I, Faculty of Medicine, University Medical Center, University of Freiburg, Freiburg, Baden-Württemberg, Germany, uni-freiburg.de; ^4^ State Key Laboratory of Medical Genomics, Shanghai Institute of Hematology, National Research Center for Translational Medicine, Shanghai Rui Jin Hospital, Shanghai Jiao Tong University School of Medicine, Shanghai, China, shsmu.edu.cn

**Keywords:** acute graft-versus-host disease, antithymocyte globulin, cytokine, haploidentical hematopoietic stem cell transplantation, prediction model

## Abstract

**Background:**

Acute graft‐versus‐host disease (aGvHD) stands as a critical complication following haploidentical hematopoietic stem cell transplantation (haplo‐HSCT). Most existing predictive models, predominantly derived from HLA‐matched donor cohorts, have been utilized for nonrelapse mortality (NRM) prediction; however, their utility in predicting aGvHD risk specifically in haplo‐HSCT recipients receiving antithymocyte globulin (ATG)–based prophylaxis warrants further validation.

**Methods:**

A total of 280 patients undergoing ATG–based haplo‐HSCT were retrospectively analyzed across two medical centers, split into training, internal test, and external validation cohorts. We first evaluated the predictive accuracy of the previously established Mount Sinai Acute GvHD International Consortium (MAGIC) algorithm for aGvHD, steroid‐refractory aGvHD (SR‐aGvHD). Subsequently, plasma concentrations of candidate cytokines (ST2, REG3α, Elafin, and TNFRI), selected a priori for their links to epithelial injury and inflammatory signaling in GvHD, were assessed for their predictive and causal relationships with aGvHD using logistic regression, weighted average area under the curve (wAUC), Mendelian randomization (MR), and restricted cubic spline (RCS) analyses. A new predictive model (the HAG model) was constructed based on identified key cytokines and validated across multicenter cohorts. MR analyses utilized external genome‐wide association (GWAS) datasets to validate the reliability of identified cytokines. A visual interface for the model was created using R *Shiny*.

**Results:**

MAGIC algorithm remains effective in the ATG–based haplo‐HSCT setting for predicting aGvHD, achieving AUC values of 0.693 (training), 0.658 (internal test), and 0.622 (external validation). Among candidate cytokines, a combination of ST2, REG3α, and Elafin (the HAG model) demonstrated the highest predictive accuracy. MR analysis leveraging external GWAS data supported potential causal associations of ST2 (OR = 1.280, *p* = 0.004), REG3α (OR = 1.300, *p* = 0.012), and Elafin (OR = 1.209, *p* = 0.039) with aGvHD risk, providing complementary biological support for their selection as candidate biomarkers. The HAG model displayed good discrimination for aGvHD (AUC = 0.636–0.701) and SR‐aGvHD (AUC = 0.666–0.779). Integration of clinical factors further enhanced prediction (HAG‐C model, wAUC from 0.682 to 0.701).

**Conclusion:**

The HAG model, incorporating ST2, REG3α, and Elafin, provides clinically meaningful prediction of aGvHD and related clinical outcomes in ATG–based haplo‐HSCT recipients, and may serve as a mechanistically informed tool for risk stratification and clinical management.

## 1. Introduction

Acute graft‐versus‐host disease (aGvHD) stands as a major complication of allogeneic hematopoietic stem cell transplantation (allo‐HSCT), contributing substantially to early non‐relapse mortality (NRM) [1]. Haploidentical HSCT (haplo‐HSCT) with T cell–modulating strategies, such as antithymocyte globulin (ATG) or posttransplant cyclophosphamide (PTCy), has broadened donor availability and reduced GvHD incidence, yet aGvHD is still observed in a significant fraction of patients [2, 3]. Moreover, aGvHD remains particularly challenging once patients become resistant to first‐line corticosteroid therapy, with overall survival (OS) reported to be only 5%–30% [4]. Early detection of high‐risk patients could enable preemptive or intensified prophylaxis and potentially improve survival [5].

In ATG–based haplo‐HSCT—notably the “Beijing Protocol” employed in approximately 94% of haploidentical transplants in China [6, 7]—the immune environment, engraftment kinetics, and immune reconstitution patterns differ from those in other settings [3], warranting additional attention. Notably, prior study developing clinical risk scores for aGvHD in haplo‐HSCT recipients undergoing ATG–based GvHD prophylaxis did not include plasma cytokines and acknowledged that incorporating biomarkers might enhance predictive performance [6]. To address the need for a reliable early warning tool in this specific context, a cytokine‐driven approach is attractive. Soluble biomarkers reflecting early tissue injury and inflammation have shown promise in stratifying the GvHD risk. In particular, the Mount Sinai Acute GvHD International Consortium (MAGIC) algorithm demonstrated that two plasma cytokines, ST2 and REG3α, can predict GvHD outcomes more accurately than clinical assessments [8–10]. However, existing cytokine algorithms have predominantly been derived from PTCy–based regimens and HLA‐matched donor cohorts [9]. Therefore, the effectiveness and accuracy of these algorithms require further validation and refinement within the ATG–based haplo‐HSCT context.

**Figure 1:**
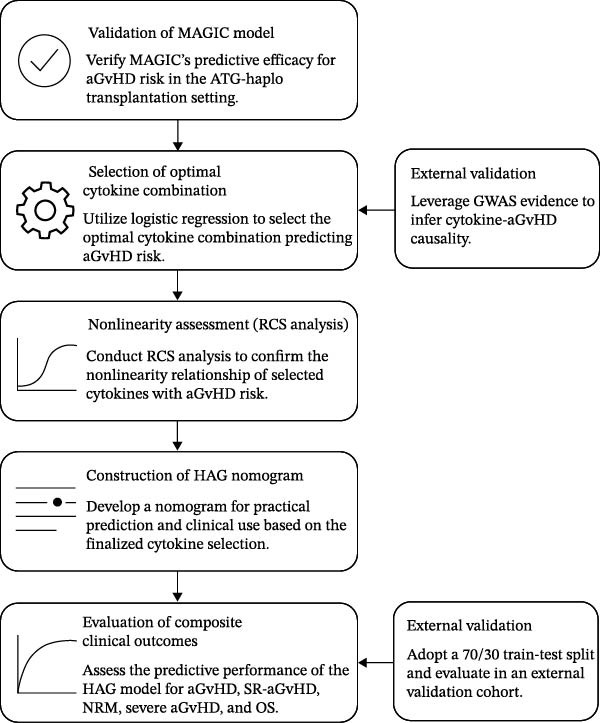
Workflow for this study. aGvHD, acute graft‐versus‐host disease; GWAS, genome‐wide association study; NRM, non‐relapse mortality; OS, overall survival; RCS, restricted cubic spline; SR‐aGvHD, steroid‐refractory acute GvHD.

In this multicenter study, we validated the performance of the MAGIC algorithm and subsequently refined it to enhance predictive capabilities specifically for aGvHD in patients undergoing ATG–based haplo‐HSCT. To further improve the rigor of feature selection, we prespecified the causal hypothesis that selected cytokines exert a causal effect on aGvHD risk and applied Mendelian randomization (MR), a method that under specific assumptions is intended to estimate causal effects, to provide orthogonal external support for the selected biomarkers. To emphasize its specificity to this clinical context, we termed this model HAG (haploidentical transplantation‐associated aGvHD). This strategy has the potential to advance precision medicine by enabling more accurate risk stratification and individualized management of ATG–based haplo‐HSCT recipients. The overall workflow of this study is illustrated in Figure 1.

## 2. Materials and Methods

### 2.1. Study Design and Patient Cohort

This study was conducted using patient data collected from two medical centers in China. The main cohort (training and internal test sets) included patients who underwent allo‐HSCT between June 2022 and September 2023 at the First Affiliated Hospital of Soochow University. The external validation cohort comprised patients transplanted at Ruijin Hospital affiliated with the Shanghai Jiao Tong University School of Medicine from October 2021 to January 2023 (NCT 06708130). Inclusion criteria were as follows: (1) age≤70 years, (2) haplo‐HSCT with ATG–based prophylaxis, and (3) available cytokine measurement within 3–20 days posttransplant prior to initiating treatment for aGvHD. Exclusion criteria included (1) severe organ dysfunction pretransplant and (2) known autoimmune diseases such as systemic lupus erythematosus (SLE) or rheumatoid arthritis (RA).

### 2.2. Causal Inference

Genome‐wide association (GWAS) summary statistics for cytokines were retrieved from a large‐scale GWAS of plasma protein levels measured with 4907 aptamers in 35,559 Icelanders [11]. SNP‐protein associations were expressed per 1‐SD increase in rank‐based inverse normal‐transformed protein levels. GWAS for outcome data for aGvHD originated from the Fred Hutchinson Cancer Research Center (FHCRC) GWAS of 4270 transplant recipients [12]. In our analyses, aGvHD was defined and assessed identically to the original FHCRC study, following the same diagnostic criteria as previously described. Independent instrumental variables (IVs) were selected with the PLINK v1.9 clump algorithm, keeping single‐nucleotide polymorphisms (SNPs) associated with each cytokine at genome‐wide significance (*p* < 5 × 10^−8^) that were in low linkage disequilibrium (*r* ^2^ < 0.001) within a 500 kb window; instrument strength was quantified by the proportion of variance explained (*r* ^2^) and the first‐stage *F*‐statistic, and variants with *F*≤10 were excluded to minimize weak‐instrument bias. For missing IVs in the outcome, we replaced them with the SNPs with the smallest *p* values within 50 kb upstream and downstream in the exposure datasets. MR rests on three core assumptions: relevance, whereby genetic instruments are robustly associated with the exposure; independence, meaning they are not related to confounders of the exposure–outcome relationship; exclusion restriction, which assumes that the variants influence the outcome only through the exposure, without alternative pathways or horizontal pleiotropy. Primary causal estimates were obtained using the inverse‐variance‐weighted (IVW) fixed‐effect method, which combines SNP‐specific Wald ratios under an assumption of valid instruments and offers optimal efficiency when all MR assumptions—relevance, independence from confounding, and exclusion restriction—are satisfied. Robustness was further evaluated with the weighted median estimator, consistent when ≥50% of the information derives from valid IVs, and the weighted mode estimator, consistent when the largest cluster of IVs is valid. Between‐instrument heterogeneity, indicative of possible assumption violation, was assessed with Cochran’s *Q* statistic for both IVW and MR‐Egger models, while directional horizontal pleiotropy was examined via the MR‐Egger intercept; when significant heterogeneity was detected, random‐effects IVW estimates were additionally reported.

### 2.3. Outcome Definitions

aGvHD was defined according to the National Institutes of Health (NIH) guidelines as an inflammatory response affecting the skin, gastrointestinal tract, or liver occurring within the first 100 days post‐HSCT. Diagnosis of aGvHD relied on clinical manifestations supported by relevant clinical examinations [13, 14]. aGvHD was graded with the modified Seattle (Glucksberg) criteria, and Grades II–IV were prespecified as severe aGvHD [15]. Steroid‐refractory aGvHD (SR‐aGvHD) was defined as aGvHD unresponsive to initial corticosteroid treatment within 28 days or requiring additional immunosuppressive therapy [8]. OS was defined as the time from allo‐HSCT to death from any cause. NRM was defined as death without prior relapse, measured from allo‐HSCT, with relapse considered a competing risk.

### 2.4. Model Development and Validation

Plasma cytokine levels, specifically ST2, REG3α, Elafin, and TNFRI, were selected a priori and measured posttransplant using an enzyme‐linked immunosorbent assay (ELISA), with detailed assay information provided in the Supporting Information. All assays were performed according to standard manufacturer protocols. Cytokine levels underwent log_10_ transformation to normalize data distributions and reduce the influence of extreme values [8]. The main cohort was randomly divided into training and test sets at a 7:3 ratio. The logistic regression model was applied to the training dataset to evaluate various combinations of cytokines (ST2, REG3α, Elafin, and TNFRI) for predicting the occurrence of aGvHD. External validation was performed using an independent cohort to evaluate the model’s generalizability and robustness. Receiver operating characteristic (ROC) curves were generated, and the area under the curve (AUC) was calculated to evaluate the model’s predictive performance in distinguishing between positive and negative cases. AUCs were reported with 95% confidence intervals (CIs) estimated by the DeLong method, and paired ROC curves were compared using DeLong’s test. Sensitivity, specificity, and accuracy were also computed to comprehensively assess the model’s diagnostic performance. An optimal threshold was determined based on the maximum Youden index in the training set. To evaluate the optimal combination, a weighted average AUC (wAUC) was calculated using the formula [16]: wAUC = *w*_train_ × AUC_train_ + *w*_test_ × AUC_test_ + *w*_val_ × AUC_val_, where *w*_train_ + *w*_test_ + *w*_val_ = 1. Elafin and TNFRI were measured in picograms per milliliter, whereas ST2 and REG3α were quantified in nanograms per milliliter. A visual interface for the model was created using R Shiny and made publicly accessible (https://ibmt.shinyapps.io/DynNomapp/). We also developed a HAG‐combined (HAG‐C) model by integrating the original HAG cytokine panel with the top four clinical features identified through random forest‐based feature selection. We further compared the performance of our models with the previously established MAGIC algorithm [4, 8]: ln[–ln(1–*p̂*)] = –11.263 + 1.844 × (log_10_ST2) + 0.577 × (log_10_REG3α), threshold = 0.160. In the MAGIC algorithm, ST2 was expressed in picograms per milliliter, and REG3α was measured in nanograms per milliliter.

### 2.5. Statistical Analysis

All statistical analyses were performed using R software (version 4.3.2) [17]. A complete case analysis was performed. Only patients with complete data for the variables included in the present analyses were retained. Patient baseline characteristics were compared with Wilcoxon rank‐sum tests and *t*‐tests for continuous variables and *x* ^2^ or corrected *x* ^2^ tests for categorical variables. For the comparison of aGvHD organ involvement and grade distributions between cohorts, binary proportions were compared using Fisher’s exact test, and the overall Grade I–IV distribution was compared using the Fisher–Freeman–Halton exact test. MR analysis was conducted with the *MendelianRandomization* package [18, 19], *mr.raps* package [20], and MR‐PRESSO was performed with the *MR-PRESSO* package [21]. A nomogram was constructed based on significant predictors to facilitate clinical interpretability and individual risk estimation. Calibration curves were used to assess the agreement between predicted probabilities and observed aGvHD incidence. In addition, quantitative calibration performance was evaluated by calculating the calibration intercept, calibration slope, and Brier score in the training, internal test, and external validation cohorts. To assess internal validity and potential overfitting, bootstrap‐based internal validation with 1000 resamples was performed for the models, and uniform shrinkage was applied using the optimism‐corrected calibration slope as the shrinkage factor. Decision curve analysis (DCA) was conducted via the R package *rmda* to assess clinical utility across various risk thresholds [22]. Nonlinear relationships between predictors and outcomes were explored using restricted cubic splines (RCS) via the R package *rms* [23]. To evaluate the potential impact of sampling time on the model performance, sensitivity analyses were performed by incorporating sampling time into models. The AUCs of the adjusted and unadjusted models were then compared using DeLong’s test in the training, internal test, and external validation cohorts. The random forest algorithm was implemented using the R package *randomForest* with default parameters for feature selection [24]. The relative importance of selected features was subsequently visualized through radar charts generated by the R package *ggradar* [25]. The *survival* package was used to test the proportional‐hazard assumption and to fit Cox proportional‐hazard regression models [26, 27]; resulting survival curves were visualized with the *survminer* [28] and *ggplot2* packages [29]. Hazard ratios (HRs), 95% CIs, and *p* values for OS were derived from Cox regression models. NRM was analyzed in a competing‐risk framework with relapse treated as a competing event, and subdistribution HRs (sHRs), 95% CIs, and *p* values were obtained from Fine–Gray regression models.

## 3. Results

### 3.1. Patient Characteristics

In total, 280 patients were included: in the training and internal test dataset, 72 patients (31.3%) developed aGvHD, while 158 patients (68.7%) did not (non‐GvHD). In the external validation cohort, 21 patients (42.0%) developed aGvHD, while 29 patients (58.0%) remained free of aGvHD. Patient baseline characteristics and sampling time are summarized in Table 1 and Supporting Information 1: Tables S1 and S2. No significant differences in common clinical characteristics were observed between patients who developed aGvHD and those who did not across both medical centers. Among patients with aGvHD, skin, liver, and gastrointestinal involvement occurred in 43/72 (59.7%), 4/72 (5.6%), and 36/72 (50.0%) cases in the training and internal test dataset and in 13/21 (61.9%), 0/21 (0.0%), and 12/21 (57.1%) cases in the external validation cohort, respectively. Grade I–IV distributions were 28/72 (38.9%), 23/72 (31.9%), 16/72 (22.2%), and 5/72 (6.9%) in the training and internal test dataset, and 7/21 (33.3%), 11/21 (52.4%), 1/21 (4.8%), and 2/21 (9.5%) in the external validation cohort, respectively. Grade II–IV aGvHD accounted for 44/72 (61.1%) and 14/21 (66.7%) cases in the training and internal test dataset and external validation cohorts, respectively (Supporting Information 1: Table S3).

**Table 1:** Clinical characteristics of the cohort.

Characteristics	Training and internal test data set			External validation data set		
	aGvHD (*n* = 72)	Non‐aGvHD (*n* = 158)	*p* value	aGvHD (*n* = 21)	Non‐aGvHD (*n* = 29)	*p* value
Age at HSCT, median (IQR)	40.5 (25, 47.75)	40 (31, 50)	0.268	51 (44, 55)	44 (25, 54)	0.175
Gender, *n* (%)	—	—	0.429	—	—	0.845
Male	45 (62.5%)	90 (57.0%)	—	11 (52.4%)	16 (55.2%)	—
Female	27 (37.5%)	68 (43.0%)	—	10 (47.6%)	13 (44.8%)	—
Blood Type, *n* (%)	—	—	0.804	—	—	0.749
A	17 (23.6%)	40 (25.3%)	—	3 (14.3%)	7 (24.1%)	—
B	21 (29.2%)	53 (33.5%)	—	9 (42.9%)	11 (38.0%)	—
O	26 (36.1%)	47 (29.7%)	—	7 (33.3%)	7 (24.1%)	—
AB	8 (11.1%)	18 (11.4%)	—	2 (9.5%)	4 (13.8%)	—
Diagnosis, *n* (%)	—	—	0.161	—	—	0.930
Leukemia	57 (79.2%)	114 (72.2%)	—	19 (90.5%)	26 (89.7%)	—
MDS, MDS/MPN	7 (9.7%)	29 (18.4%)	—	1 (4.8%)	1 (3.4%)	—
Lymphoma	4 (5.6%)	3 (1.9%)	—	1 (4.8%)	2 (6.9%)	—
Others	4 (5.6%)	12 (7.6%)	—	—	—	—
MNC infusion, median (IQR)/mean ± sd	10.206 (7.6035, 14.533)	10.518 (7.6778, 14.336)	0.950	14.016 ± 4.6869	14.195 ± 4.1561	0.888
CD34^+^ infusion, median (IQR)	4.0348 (2.8725, 5.6735)	4.0395 (3.026, 5.4437)	0.887	9.27 (8.06, 10.61)	9.58 (8.26, 10.6)	0.806
Conditioning, *n* (%)	—	—	0.289	—	—	0.702
Myeloablative	64 (88.9%)	147 (93.0%)	—	18 (85.7%)	27 (93.1%)	—
Others	8 (11.1%)	11 (7.0%)	—	3 (14.3%)	2 (6.9%)	—
GvHD prophylaxis, *n* (%)	—	—	0.677	—	—	1.000
CNI + MTX + MMF	71 (98.6%)	156 (98.7%)	—	3 (14.3%)	5 (17.2%)	—
CNI + MMF	0 (0.0%)	1 (0.6%)	—	18 (85.7%)	24 (82.8%)	—
CNI + MTX	1 (1.4%)	1 (0.6%)	—	—	—	—
PTCy use, *n* (%)	—	—	0.847	—	—	1.000
No PTCy	72 (100.0%)	156 (98.7%)	—	3 (14.3%)	5 (17.2%)	—
With PTCy	0 (0.0%)	2 (1.3%)	—	18 (85.7%)	24 (82.8%)	—
Donor gender, *n* (%)	—	—	0.761	—	—	0.390
Male	52 (72.2%)	111 (70.3%)	—	12 (57.1%)	20 (69.0%)	—
Female	20 (27.8%)	47 (29.7%)	—	9 (42.9%)	9 (31.0%)	—
Donor Blood Type, *n* (%)	—	—	0.377	—	—	0.112
A	13 (18.1%)	43 (27.2%)	—	4 (19.0%)	15 (51.7%)	—
B	24 (33.3%)	55 (34.8%)	—	6 (28.6%)	6 (20.7%)	—
O	27 (37.5%)	46 (29.1%)	—	7 (33.3%)	6 (20.7%)	—
AB	8 (11.1%)	14 (8.9%)	—	4 (19.0%)	2 (6.9%)	—
Blood Type Match, *n* (%)	—	—	0.818	—	—	0.045
Match	39 (54.2%)	83 (52.5%)	—	14 (66.7%)	11 (38.0%)	—
Mismatch	33 (45.8%)	75 (47.5%)	—	7 (33.3%)	18 (62.1%)	—
Stem cell source, *n* (%)	—	—	0.526	—	—	—
PB	59 (82.0%)	138 (87.3%)	—	21	29	—
PB + BM	12 (16.7%)	19 (12.0%)	—	—	—	—
BM	1 (1.4%)	1 (0.6%)	—	—	—	—
Donor age, median (IQR)/mean ± sd	32.5 (20.75, 42)	27.5 (19, 38.75)	0.297	34.667 ± 11.253	31.897 ± 11.194	0.393
ST2, median (IQR)	49.95 (26, 95.825)	24.6 (14.625, 45.275)	< 0.001	29.5 (20, 42.1)	28.1 (18.6, 40.1)	0.529
REG3α, median (IQR)	27.5 (16.15, 71.925)	17 (13.95, 37)	0.003	14.8 (8.5, 28.1)	8.5 (4.5, 13.3)	0.015
TNFRⅠ, median (IQR)	1640.3 (1143.9, 2269.2)	1396.3 (1093.5, 1774.2)	0.010	1800 (1477.1, 2545.6)	1640.7 (1286.5, 1876.9)	0.028
Elafin, median (IQR)	3751.7 (3367.1, 4497)	3605.8 (2350.9, 3872.6)	0.030	10,950 (7100, 17,600)	10,400 (6937.2, 12,490)	0.491

### 3.2. MAGIC Algorithm Validation

**Figure 2:**
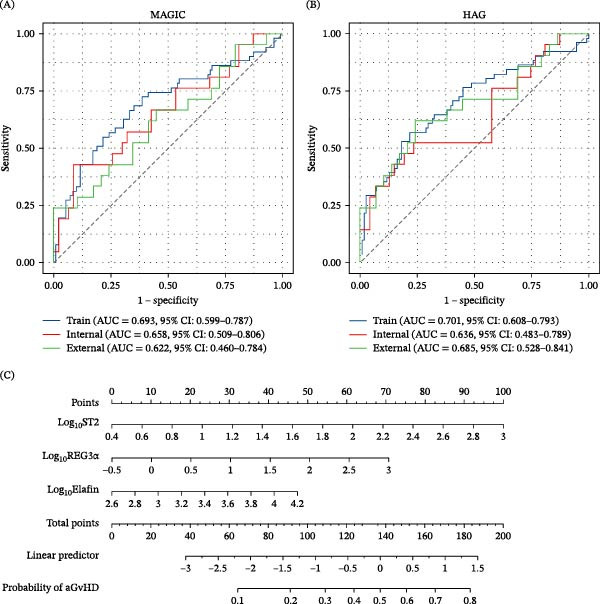
Predictive performance of cytokine models and construction of HAG prediction model. (A, B) ROC curves of the MAGIC and HAG models for predicting aGvHD in the training set, internal test set, and external validation cohort. (C) Nomogram of the HAG prediction model for estimating an individual’s probability of aGvHD. aGvHD, acute graft‐versus‐host disease.

For aGvHD prediction, the MAGIC algorithm achieved AUC values of 0.693 (95% CI: 0.599–0.787) in the training set, 0.658 (95% CI: 0.509–0.806) in the internal test set, and 0.622 (95% CI: 0.460–0.784) in the external validation cohort (Figure 2A). Regarding SR‐aGvHD prediction, MAGIC demonstrated AUC values of 0.671, 0.675, and 0.750 in the training, internal test, and external validation cohorts, respectively (Supporting Information 2: Figure S1). Collectively, these results demonstrate that the MAGIC algorithm retains acceptable predictive accuracy in patients undergoing ATG–based haplo‐HSCT.

### 3.3. Cytokine Selection and HAG Model Construction

**Figure 3:**
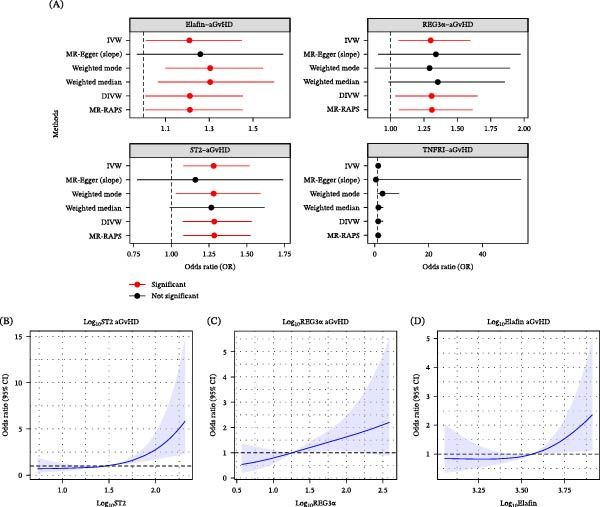
Causal‐inference screening and dose–response profiling of candidate cytokines for the HAG prediction model. (A) Forest plots summarizing MR estimates for the causal effect of genetically predicted cytokine concentrations on aGvHD. Odds ratios and 95% CIs are shown for six complementary MR methods. (B–D) Restricted cubic‐spline curves depicting the non‐linear relationship between log_10_‐transformed levels of ST2 (B), REG3α (C), and Elafin (D) and the odds of developing aGvHD. aGvHD, acute graft‐versus‐host disease; CI, confidence interval; DIVW, debiased IVW; IVW, inverse‐variance weighted; MR‐RAPS, MR‐robust adjusted profile score; OR, odds ratio.

We aimed to assess whether a context‐specific biomarker model could complement MAGIC within ATG–based haplo‐HSCT. Plasma concentrations of four candidate biomarkers (ST2, REG3α, Elafin, and TNFRI) were quantitatively analyzed in early post‐transplant samples, with all measurements being log_10_‐transformed to normalize their distributions prior to statistical evaluation. Within this predefined panel, the overall predictive performance was used as the primary criterion for biomarker selection. We evaluated all 15 possible combinations and found that the combination of ST2 + REG3α + Elafin provided the highest wAUC among the tested candidate combinations (Supporting Information 1: Table S4). Adding any additional cytokine to this trio did not appreciably improve the AUC, indicating that ST2, REG3α, and Elafin were the most informative set among the prespecified candidates (Supporting Information 1: Table S5). To explore whether this selected trio had biological relevance beyond predictive performance, we conducted two‐sample MR analyses. Fixed‐effects IVW MR demonstrated significant positive associations consistent with potential causal effects for ST2 (OR = 1.280, 95 % CI 1.080–1.517, *p* = 0.004), REG3α (OR = 1.300, 95 % CI 1.059–1.597, *p* = 0.012) and Elafin (OR = 1.209, 95 % CI 1.010–1.449, *p* = 0.039), whereas no significant association was observed for TNFRI. Alternative MR approaches—including random‐effects IVW, DIVW, MR‐RAPS, and weighted‐median estimates—yielded concordant results. The MR‐Egger intercepts were all close to zero and not significantly different from zero (all *p*  > 0.05), indicating negligible horizontal pleiotropy (Figure 3A, Supporting Information 2: Figure S2, and Supporting Information 1: Table S6). These convergent findings provide supportive genetic evidence for the biological relevance of ST2, REG3α, and Elafin in aGvHD while not establishing direct clinical causality in our cohort and lend further support to the selection of these three biomarkers as the most informative set. Therefore, the final HAG model was constructed as a logistic regression equation (Figure 2C and Table 2). The biomarker combination that achieved the highest wAUC resulted in the HAG score, calculated as: ln[–ln(1–*p̂*)] = −6.6212 + 1.1606 × (log_10_ST2) + 0.6098 × (log_10_REG3α) + 0.8937 × (log_10_Elafin). Here, *p̂* is the predicted probability of developing aGvHD. All coefficients were positive, consistent with higher cytokine levels conferring a higher risk. Additionally, RCS analyses revealed nonlinear relationships between each of the three cytokines and the risk of aGvHD (Figure 3B–D). Higher levels of *log*‐transformed ST2, REG3α, and Elafin were linked to a progressively rising risk of aGvHD, particularly at the upper range of expression. Notably, the odds of developing aGvHD increased exponentially beyond specific threshold values, with ST2 showing the most pronounced risk elevation. Using the ROC curve in the training set, we identified an optimal risk cut‐off of 0.364 for the HAG score according to the Youden Index. Patients with *p̂* ≥ 0.364 were classified as high‐risk for aGvHD. A web‐based interactive visualization tool for the model is made publicly available at https://ibmt.shinyapps.io/DynNomapp/.

**Table 2:** Univariate and multivariable logistic regression analyses of cytokines predicting aGvHD risk.

Variable	Univariate						Multivariable					
	Beta	SE	OR	95% CI lower	95% CI upper	*p* value	Beta	SE	OR	95% CI lower	95% CI upper	*p* value
log_10_ST2	1.426	0.414	4.164	1.851	9.366	0.001	1.161	0.438	3.192	1.354	7.527	0.008
log_10_REG3α	0.881	0.317	2.414	1.296	4.496	0.005	0.610	0.341	1.840	0.943	3.590	0.074
log_10_Elafin	0.966	0.743	2.627	0.613	11.263	0.193	0.894	0.762	2.444	0.549	10.876	0.241

### 3.4. Predictive Performance of the HAG Model

The HAG model demonstrated good discriminative performance across all datasets. In the training set, the AUC was 0.701 (95% CI: 0.608–0.793), indicating a fair ability to distinguish patients who developed aGvHD from those who did not. At the predefined threshold of 0.364, the sensitivity and specificity were 56.9% and 78.4%, respectively, with an overall accuracy of 71.6%. When evaluated in the internal test set, the HAG model yielded an AUC of 0.636 (95% CI: 0.483–0.789), reflecting moderate discrimination. Sensitivity decreased to 42.9%, but specificity improved slightly to 80.9%, maintaining an accuracy of 69.1%. In the external validation cohort, the HAG model achieved an AUC of 0.685 (95% CI: 0.528–0.841), with sensitivity and specificity at 61.9% and 75.9%, respectively, and an accuracy of 70.0% (Figure 2B). The wAUC combining the training, internal test, and external validation cohorts was approximately 0.68, demonstrating the model’s overall stable predictive capability across different patient groups (Supporting Information 1: Table S4). DCA showed that the HAG model achieved a favorable net benefit over the treat‐all and treat‐none strategies across the training, internal test, and external validation cohorts, particularly at lower to intermediate threshold probabilities, with a broadly consistent pattern across datasets (Supporting Information 2: Figure S3). The calibration analysis indicated an agreement between the predicted and observed aGvHD incidence (Supporting Information 2: Figure S4 and Supporting Information 1: Table S7). DeLong’s test showed no significant difference in AUC between the HAG and MAGIC models across cohorts, which may be related to the limited sample size and event number (Supporting Information 1: Table S8). Sampling time did not differ significantly between controls and aGvHD cases within either the development dataset or the external validation cohort, although it differed between cohorts (Supporting Information 1: Table S2). Adjustment for sampling time resulted in only minimal changes in AUC for both the biomarker‐based model and the HAG model, and DeLong tests showed no significant differences between the unadjusted and adjusted models across the training, internal test, and external validation cohorts (Supporting Information 2: Figure S5 and Supporting Information 1: Table S9). These findings suggested that the inclusion of Elafin may improve predictive accuracy in the ATG–based haplo‐HSCT setting through its combined effect with other biomarkers.

Additionally, we developed the HAG‐C model by integrating the top‐ranked clinical predictors identified through random forest analysis. The radial variable importance plot revealed the following key contributors: CD34^+^ infusion (mean decrease gini = 5.92), recipient age (4.97), mononuclear cell (MNC) infusion (4.84), and donor age (4.58; Supporting Information 2: Figure S6 and Supporting Information 1: Table S10). Incorporation of recipient age and CD34^+^ cell dose into the HAG framework increased the wAUC from 0.682 to 0.701, yielding a relative improvement of 2.8% in model performance (Supporting Information 1: Table S11).

To further assess potential overfitting, we performed bootstrap‐based internal validation with 1000 resamples for both the HAG and HAG‐C models. For the HAG model, the apparent AUC was 0.701, with a mean optimism of 0.022, yielding an optimism‐corrected AUC of 0.679. For the HAG‐C model, the apparent AUC was 0.738, the mean optimism was 0.021, and the optimism‐corrected AUC was 0.717. The optimism‐corrected calibration slopes were 0.908 for HAG and 0.910 for HAG‐C (Supporting Information 1: Table S12). Uniform shrinkage was subsequently applied using the optimism‐corrected calibration slope as the shrinkage factor, followed by reestimation of the intercept. After shrinkage, regression coefficients were modestly attenuated, whereas the direction and relative contribution of predictors were preserved (Supporting Information 1: Table S13). Notably, the HAG‐C model retained better discrimination than the HAG model after optimism correction, supporting the incremental predictive value of the added clinical variables.

We further examined the predictive performance of the HAG model in identifying patients with SR‐aGvHD (Supporting Information 2: Figure S1). For SR‐aGvHD prediction, the HAG model demonstrated AUC values of 0.666 in the training set, 0.688 in the internal test set, and 0.779 in the external validation cohort.

**Figure 4:**
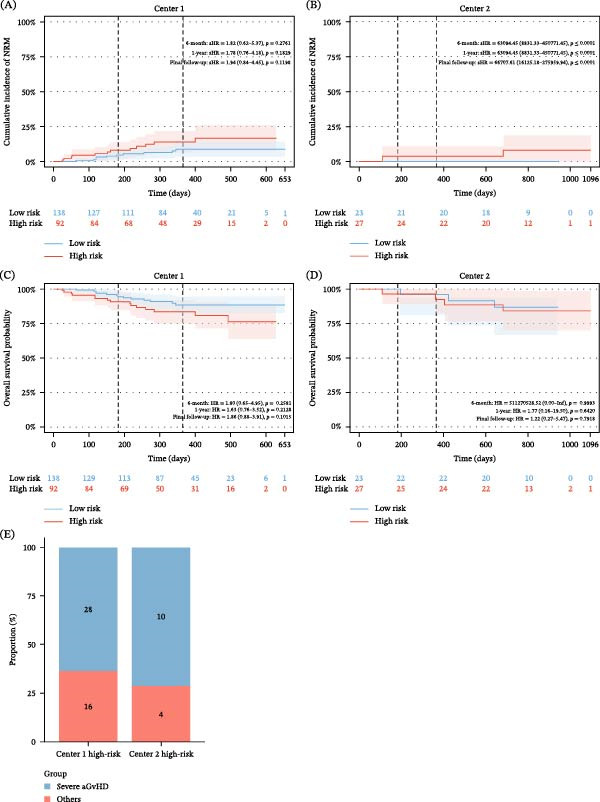
HAG model‐based risk stratification for NRM, OS, and severe aGvHD across two centers. (A, B) Cumulative incidence of non‐relapse mortality in Center 1 (A) and Center 2 (B), stratified by HAG‐defined low‐risk and high‐risk groups. (C, D) Kaplan–Meier estimates of overall survival in Center 1 (C) and Center 2 (D), stratified by HAG‐defined low‐risk and high‐risk groups. (E) Stacked bar chart showing the proportion of severe acute graft‐versus‐host disease (aGvHD) versus other outcomes among HAG high‐risk recipients with aGvHD in Center 1 and Center 2. aGvHD, acute graft‐versus‐host disease; HR, hazard ratio; sHR, subdistribution hazard ratio.

Among recipients stratified by the HAG model, NRM, OS, and the proportion of severe aGvHD were evaluated separately in the training and internal test cohort (Center 1: The First Affiliated Hospital of Soochow University) and the external validation cohort (Center 2: Ruijin Hospital, Shanghai Jiao Tong University School of Medicine; Figure 4A–D). In Center 1, high‐risk recipients showed numerically higher cumulative incidence of NRM and poorer OS than low‐risk recipients, although the corresponding sHRs and HRs were not statistically significant. In Center 2, high‐risk recipients exhibited a substantially higher cumulative incidence of NRM, whereas OS remained broadly similar between the two risk groups (Figure 4A–D). Among high‐risk recipients who developed aGvHD, severe aGvHD (Glucksberg Grades II–IV) occurred in 63.6% (28/44) of patients in Center 1 and 71.4% (10/14) in Center 2 (Figure 4E).

## 4. Discussion

In this study, we initially validated the performance of the MAGIC algorithm in patients undergoing ATG–based haplo‐HSCT and subsequently developed the HAG model as a context‐specific candidate model. To our knowledge, this represents the first cytokine‐based predictive algorithm specifically tailored for the ATG–based haplo‐HSCT setting—a transplantation context that is widely employed yet insufficiently addressed by prior biomarker studies. By integrating plasma levels of ST2, REG3α, and Elafin, the HAG model demonstrated reasonably good performance in distinguishing patients at high risk of developing aGvHD from those likely to remain disease‐free, and it further proved capable of predicting a variety of additional post‐transplant clinical outcomes. These findings offer a meaningful refinement of existing risk‐stratification methods within this clinical scenario.

The HAG model demonstrated predictive performance broadly comparable to that of the established MAGIC algorithm, suggesting that cohort‐specific biomarker refinement may provide incremental predictive value in selected settings. In earlier multi‐center analyses of heterogeneous transplant populations, adding Elafin to the ST2 + REG3α panel yielded minimal gains [30, 31]. For example, Ferrara et al. reported an AUC of 0.74 with ST2 + REG3α versus 0.76 with ST2 + REG3α + Elafin for predicting 6‐month NRM, a difference that was not statistically significant. However, in our ATG–based haplo‐HSCT cohort, inclusion of Elafin led to an increase in predictive power in the external validation cohort (from AUC 0.642–0.685, 6.7%), suggesting that Elafin provides meaningful additional information in this specific setting. One explanation is that our endpoint was the occurrence of any grade of aGvHD, which includes cases of predominant skin involvement where Elafin is particularly pertinent [32, 33].

Consistent with the MAGIC algorithm, cytokine‐based risk‐assignment models outperformed established clinical aGvHD risk factors—including the conditioning regimen, HLA mismatch degree, and donor relationship. Notably, incorporating these clinical variables into cytokine models did not significantly improve the predictive performance [8]. Nevertheless, we have provided an additional model incorporating clinical variables (HAG‐C), which may allow for further comparisons of predictive accuracy between purely cytokine‐driven (HAG) and combined clinical‐cytokine (HAG‐C) approaches in future studies.

Our findings reinforce the concept that biomarker panels must be tailored to the transplant context for optimal performance. This context‐dependent variability was exemplified by ST2 thresholds predictive of NRM, where 14‐day post‐HCT concentrations diverged markedly between reduced‐intensity (300 pg/mL) and myeloablative TBI–based regimens (1660 pg/mL) [34]. Similarly, ATG depletes T cells pretransplant, leading to delayed T‐cell recovery and perhaps a different cytokine milieu [35, 36]. It is conceivable that certain inflammatory pathways play a relatively larger role in aGvHD that occurs despite ATG, making an injury marker like Elafin more valuable. Interestingly, our work aligns with some prior observations in related settings: in T cell–depleted transplants using ATG or alemtuzumab, a broader panel including Elafin was shown to predict aGvHD severity [37]. These reports lend support to the biological plausibility of our chosen biomarker trio.

Although the HAG model retained predictive utility in the external validation cohort, its performance was more modest than that in the derivation/internal cohorts, and the quantitative calibration metrics suggested some deviation from ideal calibration. A likely explanation is the substantial inter‐center heterogeneity between cohorts, particularly in GvHD prophylaxis. The derivation/internal cohort predominantly received CNI + MTX + MMF, whereas PTCy–based prophylaxis was much more common in the external cohort. Because prophylactic strategies can influence posttransplant immune reconstitution, inflammatory signaling, and tissue injury [38], such differences may affect circulating biomarker profiles and model performance across centers [39]. Although subgroup analysis within the external cohort did not show statistically significant differences in cytokine levels according to PTCy use (Supporting Information 2: Figure S7), this finding should be interpreted cautiously given the limited sample size. Consistent with this, biomarker behavior was not uniform across cohorts: TNFRI remained significant across centers, and REG3α retained significance in the external cohort, whereas ST2 and Elafin did not. These findings suggest that intercohort heterogeneity may affect individual biomarkers unequally and that their relative contributions may depend on the transplant platform, prophylaxis composition, and organ involvement. Sampling time may represent an additional source of variation. Although sampling time did not differ significantly between controls and aGvHD cases within either cohort, its distribution differed between the development and external validation cohorts. However, adjustment for sampling time resulted in only minimal changes in AUC for both the biomarker‐based model and the HAG model, and DeLong testing showed no significant differences between the unadjusted and adjusted models, suggesting that sampling‐time variation did not materially account for the observed differences in discrimination. Therefore, while the HAG model supports the value of biomarker‐based risk stratification in the ATG–based haplo‐HSCT setting, its performance and the contribution of individual markers should be interpreted in the context of center‐ and regimen‐specific differences.

From a clinical perspective, the HAG model could enable preemptive aGvHD management. Patients identified as high‐risk (elevated HAG score) soon after transplantation could be monitored more closely or considered for prophylactic interventions. Conversely, low‐risk patients might be spared unnecessary early interventions and could be candidates for careful tapering of immunosuppression. This risk‐adapted approach is analogous to ongoing strategies where biomarker‐guided therapy is being tested [40–42]. The availability of an easy‐to‐use web calculator for HAG enhances its potential clinical translatability.

Several limitations are acknowledged in this study. First, the current dataset for cytokine analysis in our ATG–based haplo‐HSCT cohort is limited. Further research involving larger patient populations will be necessary to validate and potentially improve the model’s predictive performance for aGvHD. Additionally, as more patient data becomes available, particularly concerning aGvHD events, we intend to continue evaluating the utility of our prediction model for important outcomes such as NRM and Grade II–IV aGvHD. Second, biomarker selection was restricted to four prespecified, literature‐supported candidates. This approach enhanced feasibility and interpretability but did not allow unbiased discovery of all potentially informative cytokines or proteomic markers. In addition, differences in the transplant platform and GvHD prophylaxis between the derivation/internal and external validation cohorts may have contributed to variability in biomarker performance, which should be taken into account when interpreting the generalizability of the HAG model. Moreover, the MR analyses were based on external GWAS and proteomic datasets derived from Icelandic populations and, therefore, may not be directly generalizable to our cohort. Population differences in the genetic background, protein quantitative trait loci, transplant‐related exposures, and immune context may influence the transferability of these findings. Furthermore, although MR can strengthen the causal inference under specific assumptions, it does not establish direct clinical causality in this setting. Accordingly, the MR results should be interpreted as supportive evidence for biological plausibility rather than definitive proof of causality in our cohort.

## 5. Conclusion

In conclusion, we first confirmed the predictive utility of the MAGIC algorithm in patients undergoing ATG–based haplo‐HSCT and subsequently built upon this foundation to create the HAG model. The HAG model incorporates ST2, REG3α, and Elafin, which emerged as the most informative biomarkers among the prespecified candidates evaluated in this study. The availability of a publicly accessible online platform further enhances the clinical applicability and ease of implementation of this predictive tool. Future prospective trials are warranted to validate these results, ultimately supporting its role in personalized risk‐adaptive management strategies.

## Author Contributions

Yang Xu, Yiyin Chen, Xinghao Yu, and Xiaoxia Hu designed the study. Yiyin Chen, Xinghao Yu, Zhou Jin, and Chuanhe Jiang collected the data. Yiyin Chen, Xinghao Yu, Zhou Jin, and Chuanhe Jiang curated and cleaned the datasets. Yiyin Chen, Xinghao Yu, Zhou Jin, and Chuanhe Jiang mainly performed the data analyses. Yiyin Chen, Xinghao Yu, Zhou Jin, Chuanhe Jiang, Yang Xu, and Xiaoxia Hu drafted the manuscript. Yang Xu, Xiaoxia Hu, Yiyin Chen, Xinghao Yu, Zhou Jin, and Chuanhe Jiang revised the manuscript.

## Funding

Yang Xu is supported by the Noncommunicable Chronic Diseases‐National Science and Technology Major Project (Grant 2025ZD0545800/2025ZD0545803), the National Natural Science Foundation of China (Grants U25A2013 and 82070187), and Priority Academic Program Development of Jiangsu Higher Education Institutions (PAPD). Xiaoxia Hu is supported by the National Key Research and Development Program (Grant 2022YFC2502600) and the National Natural Science Foundation of China (Grants 82470213 and 82170206). Xinghao Yu is supported by the Noncommunicable Chronic Diseases‐National Science and Technology Major Project (Grant 2024ZD0534700), the Suzhou Science and Technology Program Project (Grant QNXM2024010), the Suzhou Basic Research Youth Special Project (Grant SSD2025070), and the Jiangsu Provincial Health Commission Project (Grant MQ2024022). Zhou Jin is supported by Prof. Changgeng Ruan’s Research and Innovation Fund for Graduate Students, the First Affiliated Hospital of Soochow University.

## Disclosure

All authors have read and approved the final manuscript.

## Ethics Statement

This study was approved by the Ethics Committees of The First Affiliated Hospital of Soochow University (Approval Number 2024774) and Ruijin Hospital affiliated with Shanghai Jiao Tong University School of Medicine (Approval Number 202408).

## Consent

The authors have nothing to report.

## Conflicts of Interest

The authors declare no conflicts of interest.

## Supporting Information

Additional supporting information can be found online in the Supporting Information section.

## Supporting information


**Supporting Information 1** Table S1: Distribution of sampling time from transplantation to biomarker measurement. Table S2: Statistical comparisons of sampling time between cohorts and aGvHD outcome groups. Table S3: aGvHD organ involvement at treatment initiation and overall grade distribution among patients who developed aGvHD. Table S4: Predictive performance of cytokine combinations. Table S5: Weighted average AUCs of cytokine combinations. Table S6: Causal associations between cytokines and aGvHD estimated using complementary Mendelian randomization methods. Table S7: Quantitative calibration metrics of the HAG model in the training, internal test, and external validation cohorts. Table S8: DeLong test results comparing the predictive performance of the MAGIC and HAG models across datasets. Table S9: DeLong test comparing model discrimination before and after adjustment for sampling time in the training, internal test, and external validation. Table S10: Random forest feature importance for aGvHD prediction. Table S11: Predictive performance of clinical characteristic and HAG combinations. Table S12: Bootstrap‐based internal validation and uniform shrinkage of the HAG and HAG‐C models. Table S13: Original and shrinkage‐adjusted coefficients for the HAG and HAG‐C models.

**Supporting Information 2** Figure S1: ROC curves of the MAGIC and HAG models for predicting SR‐aGvHD in the training set, internal test set, and external validation cohort. SR‐aGvHD, steroid‐refractory acute graft‐versus‐host disease. Figure S2: Mendelian randomization assessment of cytokine levels and risk of aGvHD. (A–D) Scatter plots display the per‐allele effect of each SNP on ST2 (A), Elafin (B), REG3α (C), and TNFRI (D) concentrations (*x*‐axis) versus the corresponding log‐odds of developing aGvHD (*y*‐axis). (E–H) Funnel plots assessing heterogeneity and directional pleiotropy for the same cytokines. aGvHD, acute graft‐versus‐host disease; IVW, inverse‐variance weighted; SE, standard error. Figure S3: Decision curve analysis of the HAG model in the training, internal test, and external validation cohorts. DCA was used to evaluate the clinical utility of the HAG model for aGvHD risk stratification across three cohorts. The red line denotes the standardized net benefit of the HAG model, while the gray lines denote the treat‐all and treat‐none strategies. Threshold probability is shown on the *x*‐axis, and standardized net benefit is shown on the *y*‐axis. aGvHD, acute graft‐versus‐host disease; DCA, decision curve analysis. Figure S4: Calibration Analysis of the HAG Model for aGvHD risk prediction across cohorts. Calibration curves comparing predicted probabilities (*x*‐axis) against observed event frequencies (*y*‐axis) for the HAG model. Solid blue lines represent LOESS fits of model predictions, with gray shaded areas indicating 95% confidence intervals derived from local regression standard errors. The red dashed line (*y* = *x*) denotes ideal calibration. aGvHD, acute graft‐versus‐host disease; LOESS, locally weighted scatterplot smoothing. Figure S5: Sensitivity analyses adjusting for sampling time in the training, internal test, and external validation cohorts. Receiver operating characteristic (ROC) curves comparing the unadjusted models and models additionally adjusted for sampling time are shown for the HAG model (A–C) and the biomarker model (D–F) across the training, internal test, and external test datasets. The HAG model represents the composite HAG score used as a single predictor, whereas the biomarker model includes the three individual component biomarkers (log‐transformed ST2, REG3α, and Elafin) entered jointly in the regression model. Figure S6: Radar chart of multivariable risk factors for aGvHD. aGvHD, acute graft‐versus‐host disease; BM, bone marrow; CNI, calcineurin inhibitor; MDS, myelodysplastic neoplasms; MMF, mycophenolate mofetil; MNC, mononuclear cells; MPN, myeloproliferative neoplasm; MTX, methotrexate; PB, peripheral blood. Figure S7: Comparison of cytokine levels according to PTCy use in the external validation cohort. PTCy, post‐transplant cyclophosphamide.

## Data Availability

The GVHD data used for these analyses were obtained with permission from the FHCRC. The data for blood proteins are available in public, open‐access repositories corresponding to the original studies (e.g., the GWAS Catalog). The remaining data that support the findings of this study are available from the corresponding author upon reasonable request.
