# Supplementary material for: Development and Validation of a Cytokine‐Based Predictive Model for Acute GvHD and Composite Outcomes in ATG–Based Haploidentical Hematopoietic Stem Cell Transplantation
Source: Mediators Inflamm. 2026 Jun 30;2026:3165406. doi: 10.1155/mi/3165406 (PMC13317467; doi:10.1155/mi/3165406)
Supplement: Supplementary file 2 — Supporting Information 2 Figure S1: ROC curves of the MAGIC and HAG models for predicting SR‐aGvHD in the training set, internal test set, and external validation cohort. SR‐aGvHD, steroid‐refractory acute graft‐versus‐host disease. Figure S2: Mendelian randomization assessment of cytokine levels and risk of aGvHD. (A–D) Scatter plots display the per‐allele effect of each SNP on ST2 (A), Elafin (B), REG3α (C), and TNFRI (D) concentrations (x‐axis) versus the corresponding log‐odds of developing aGvHD (y‐axis). (E–H) Funnel plots assessing heterogeneity and directional pleiotropy for the same cytokines. aGvHD, acute graft‐versus‐host disease; IVW, inverse‐variance weighted; SE, standard error. Figure S3: Decision curve analysis of the HAG model in the training, internal test, and external validation cohorts. DCA was used to evaluate the clinical utility of the HAG model for aGvHD risk stratification across three cohorts. The red line denotes the standardized net benefit of the HAG model, while the gray lines denote the treat‐all and treat‐none strategies. Threshold probability is shown on the x‐axis, and standardized net benefit is shown on the y‐axis. aGvHD, acute graft‐versus‐host disease; DCA, decision curve analysis. Figure S4: Calibration Analysis of the HAG Model for aGvHD risk prediction across cohorts. Calibration curves comparing predicted probabilities (x‐axis) against observed event frequencies (y‐axis) for the HAG model. Solid blue lines represent LOESS fits of model predictions, with gray shaded areas indicating 95% confidence intervals derived from local regression standard errors. The red dashed line (y = x) denotes ideal calibration. aGvHD, acute graft‐versus‐host disease; LOESS, locally weighted scatterplot smoothing. Figure S5: Sensitivity analyses adjusting for sampling time in the training, internal test, and external validation cohorts. Receiver operating characteristic (ROC) curves comparing the unadjusted models and models additionally adjusted [file MI-2026-3165406-s001.docx]

# Supplementary Figures


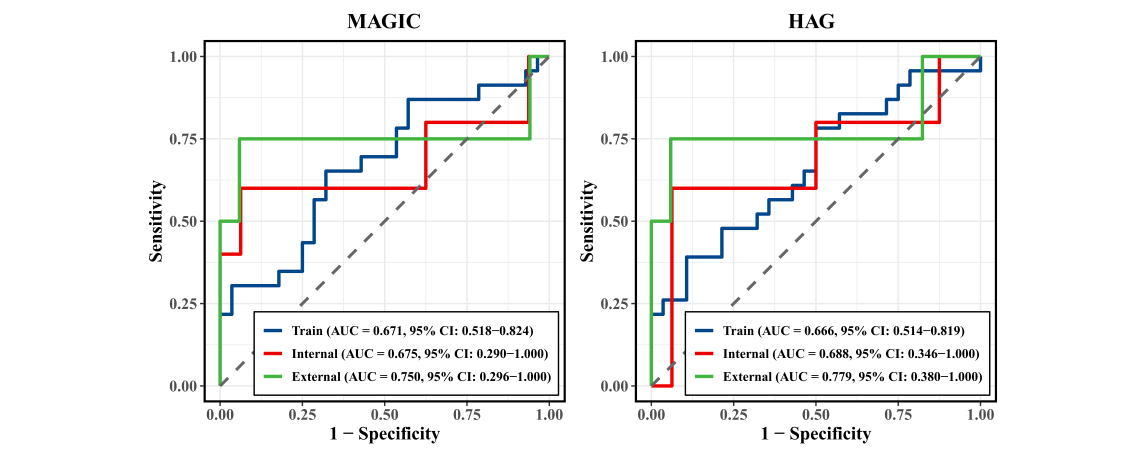


**Figure S1. ROC curves of the MAGIC and HAG models for predicting SR-aGvHD in the training set, internal test set, and external validation cohort.** Abbreviations: SR-aGvHD, Steroid-refractory acute graft-versus-host disease.


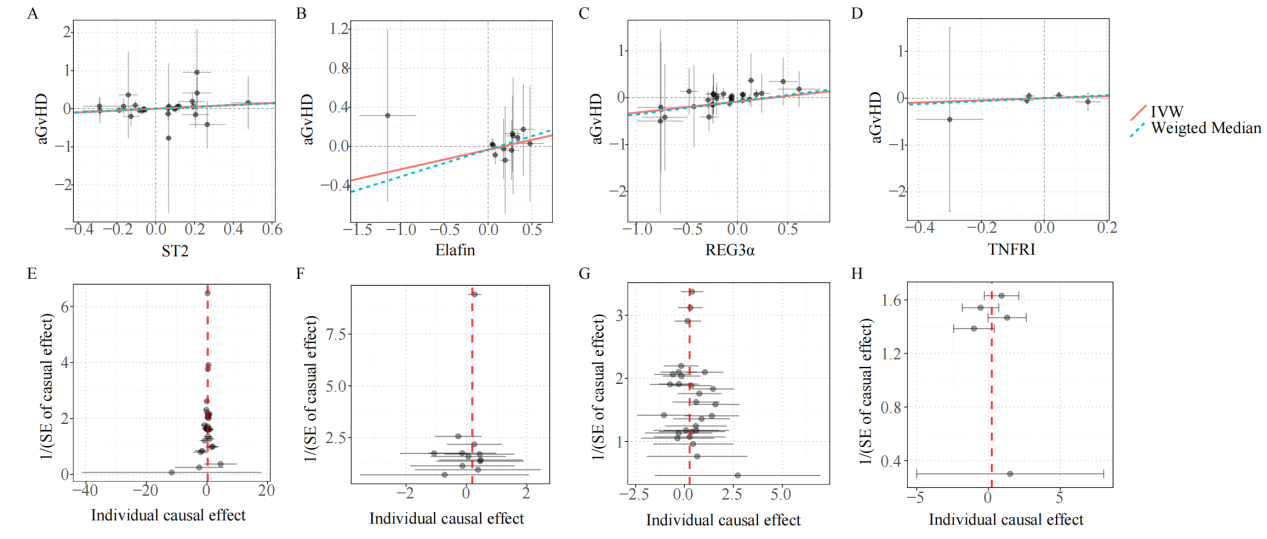


**Figure S2. Mendelian-randomization Assessment of Cytokine Levels and Risk of aGvHD.** (A-D) Scatter plots display the per-allele effect of each SNP on ST2 (A), Elafin (B), REG3α (C) and TNFRⅠ (D) concentrations (x-axis) versus the corresponding log-odds of developing aGvHD (y-axis). (E-H) Funnel plots assessing heterogeneity and directional pleiotropy for the same cytokines. Abbreviations: aGvHD, acute graft-versus-host disease; SE, standard error; IVW, inverse-variance weighted.

**
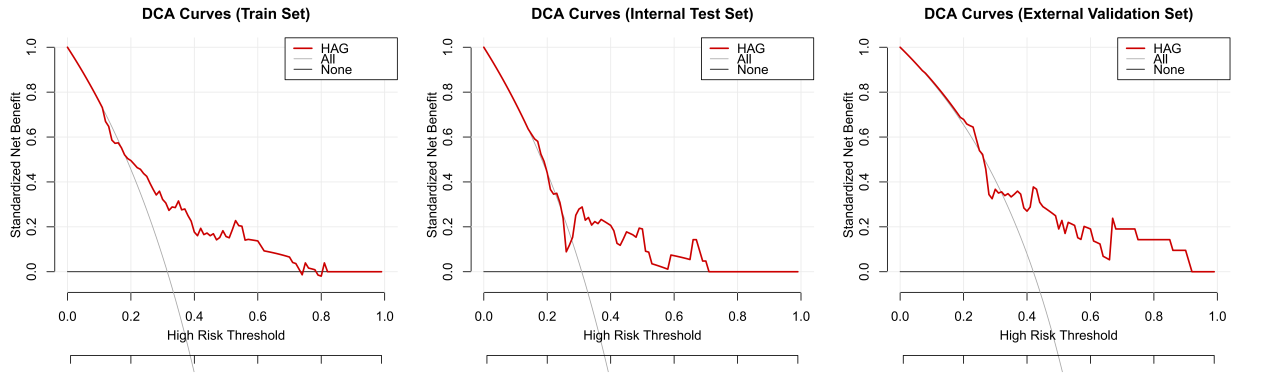
**

**Figure S3. Decision curve analysis of the HAG model in the training, internal test, and external validation cohorts.** DCA was used to evaluate the clinical utility of the HAG model for aGvHD risk stratification across three cohorts. The red line denotes the standardized net benefit of the HAG model, while the grey lines denote the treat-all and treat-none strategies. Threshold probability is shown on the x-axis, and standardized net benefit is shown on the y-axis. Abbreviations: aGvHD, acute graft-versus-host disease; DCA, decision curve analysis.

**
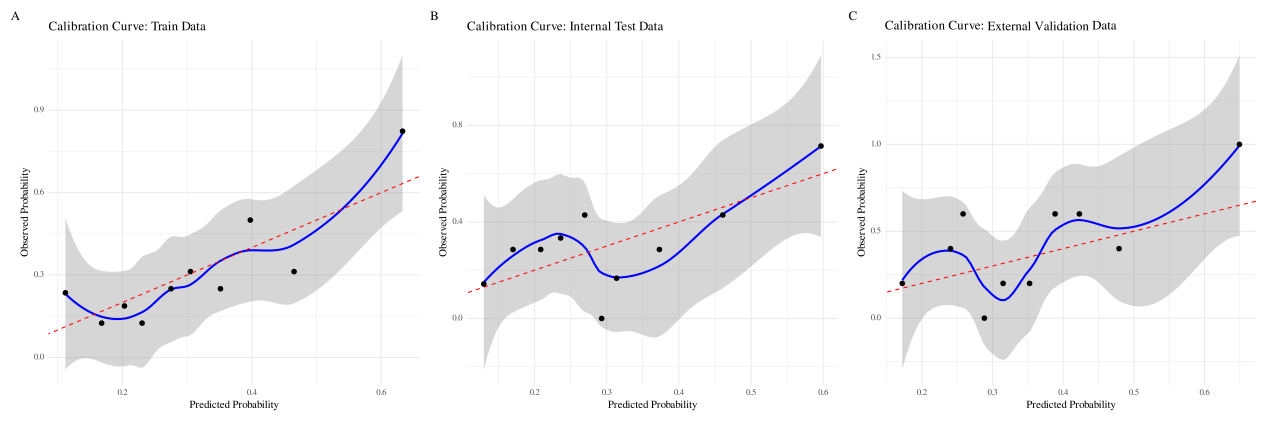
**

**Figure S4. Calibration Analysis of the HAG Model for aGvHD Risk Prediction Across Cohorts.** Calibration curves comparing predicted probabilities (x-axis) against observed event frequencies (y-axis) for the HAG model. Solid blue lines represent LOESS fits of model predictions, with gray shaded areas indicating 95% confidence intervals derived from local regression standard errors. The red dashed line (y=x) denotes ideal calibration. Abbreviations: aGvHD, acute graft-versus-host disease; LOESS, locally weighted scatterplot smoothing.


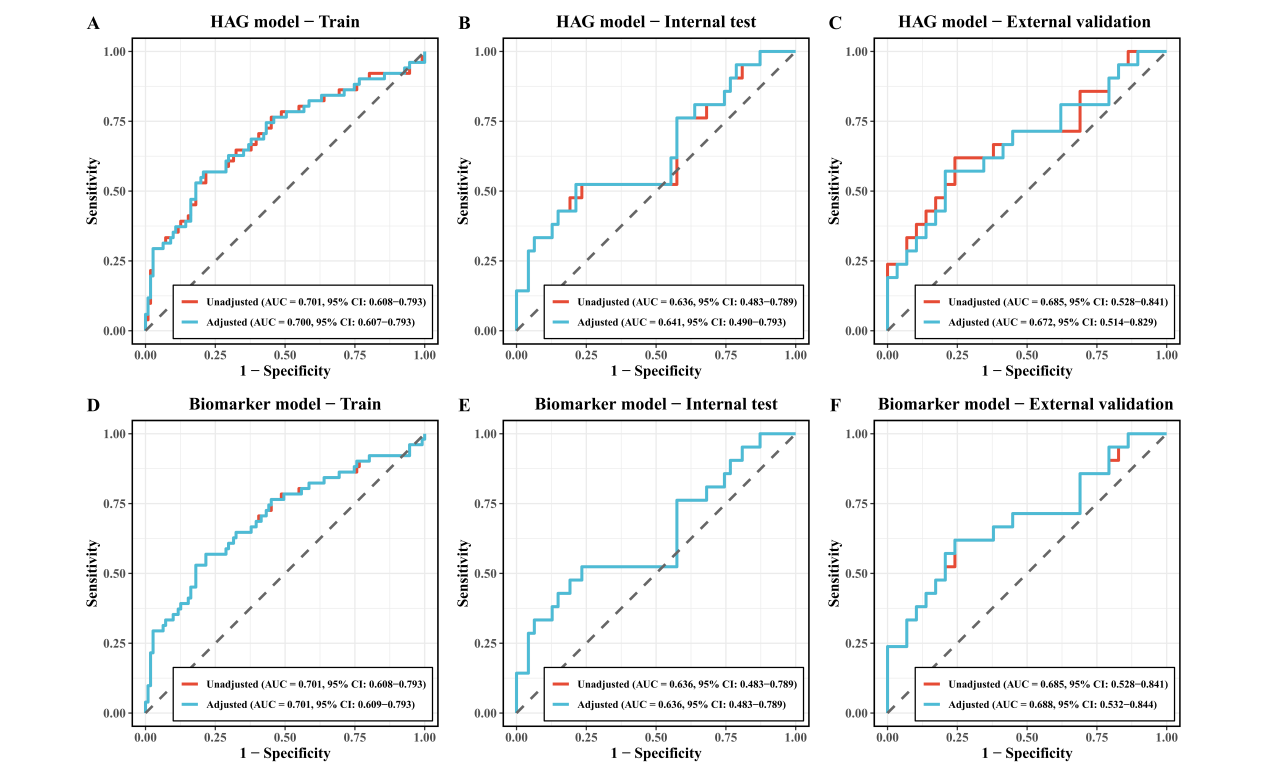


**Figure S5. Sensitivity analyses adjusting for sampling time in the training, internal test, and external validation cohorts.**

Receiver operating characteristic (ROC) curves comparing the unadjusted models and models additionally adjusted for sampling time are shown for the HAG model (A-C) and the biomarker model (D-F) across the training, internal test, and external test datasets. The HAG model represents the composite HAG score used as a single predictor, whereas the biomarker model includes the three individual component biomarkers (log-transformed sST2, REG3α, and Elafin) entered jointly in the regression model.


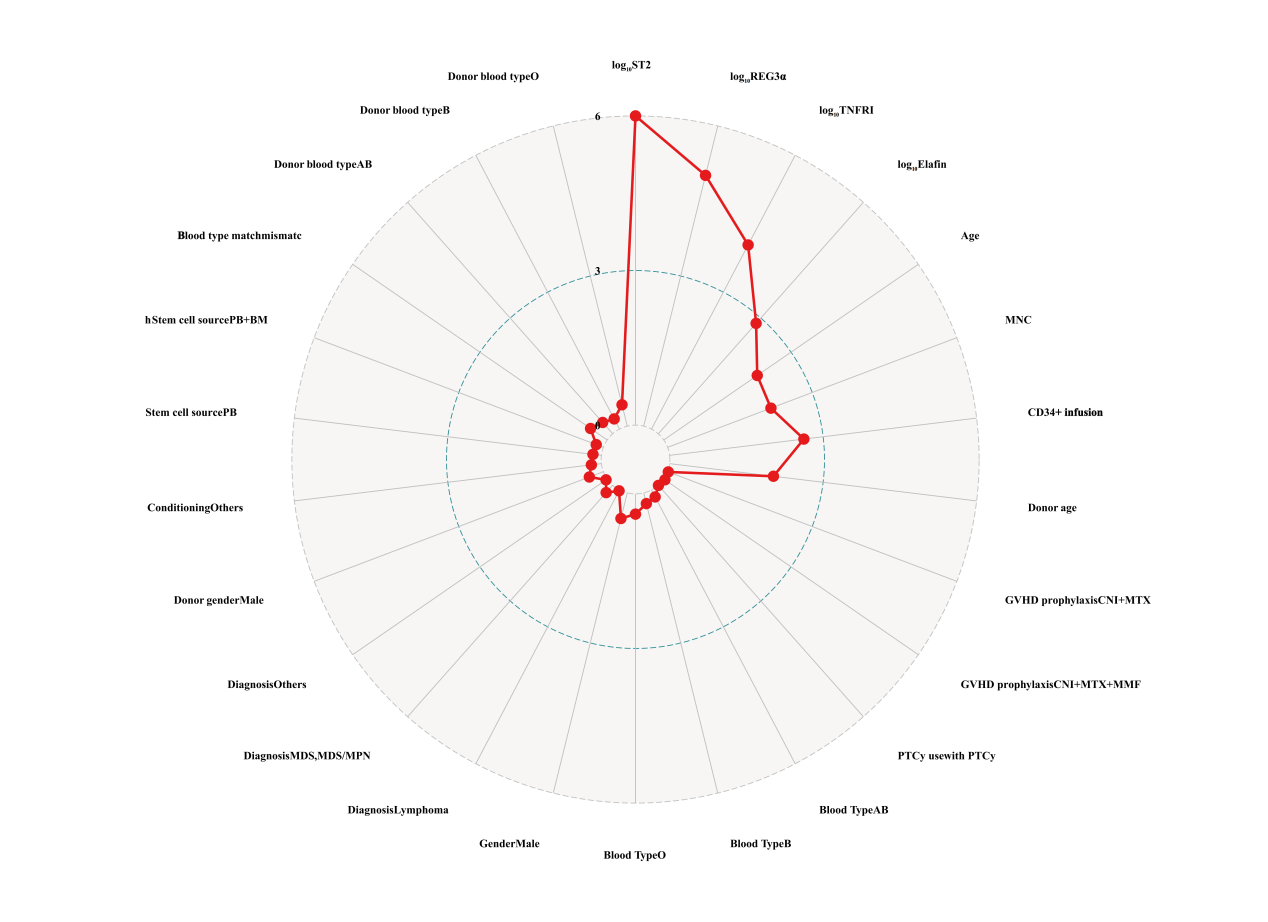


**Figure S6. Radar Chart of Multivariable Risk Factors for aGvHD.** Abbreviations: aGvHD, acute graft-versus-host disease; MNC, mononuclear cells; PB, peripheral blood; BM, bone marrow; CNI, calcineurin inhibitor; MTX, methotrexate; MMF, mycophenolate mofetil; MDS, myelodysplastic neoplasms; MPN, myeloproliferative neoplasm.

**
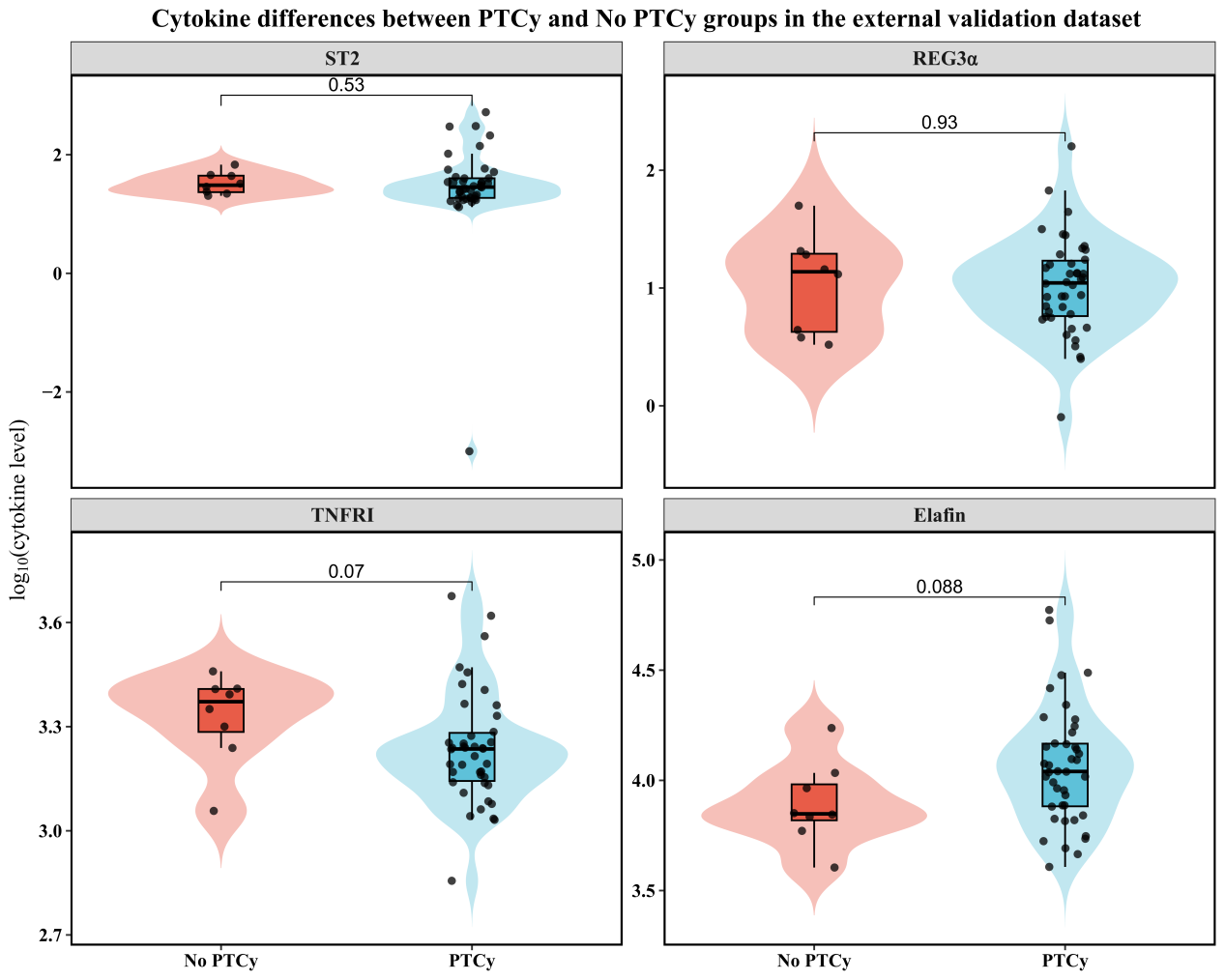
**

**Figure S7. Comparison of cytokine levels according to PTCy use in the external validation cohort.** Abbreviations: PTCy, post-transplant cyclophosphamide.

**Supplementary Methods**

**Cytokine quantification**

Peripheral blood was collected after transplantation, plasma was isolated by centrifugation, and aliquots were stored at -80°C until analysis. Plasma ST2, REG3α, TNFRI, and Elafin concentrations were measured by ELISA according to the manufacturers’ instructions. ST2 was measured using the Human ST2/IL-33R Quantikine ELISA Kit (R&D Systems, DST200; assay range 31.3-2000 pg/mL; MDD 2.45-13.5 pg/mL, mean 5.1 pg/mL; intra-assay CV 4.4%-5.6%; inter-assay CV 5.4%-7.1%). TNFRI was measured using the Human TNF RI/TNFRSF1A Quantikine ELISA Kit (R&D Systems, DRT100; assay range 7.8-500 pg/mL; MDD 0.43-1.20 pg/mL, mean 0.77 pg/mL; serum/plasma intra-assay CV 3.6%-5.0%; inter-assay CV 3.7%-8.8%). REG3α was measured using the Ab-Match Assembly Human PAP1 (REG3α) Kit (MBL, 5323) in combination with the Ab-Match Universal Kit (MBL, 5310); the manufacturer reports an LOB of 0.24 ng/mL, an LOQ of 3.41 ng/mL, and intra-assay CVs of 3.2%-8.9%, with a calibration range extending to approximately 100 ng/mL. Elafin was measured using the Human Trappin-2/Elafin DuoSet ELISA (R&D Systems, DY1747; assay range 31.2-2000 pg/mL).
